# The Role of Dydrogesterone in the Management of Luteal Phase Defect: A Comprehensive Review

**DOI:** 10.7759/cureus.48194

**Published:** 2023-11-03

**Authors:** Shaikh Muneeba, Neema Acharya, Shazia Mohammad

**Affiliations:** 1 Obstetrics and Gynecology, Jawaharlal Nehru Medical College, Datta Meghe Institute of Higher Education and Research, Wardha, IND

**Keywords:** endometrial receptivity, combination therapy, progestogen, reproductive outcomes, luteal phase defect, dydrogesterone

## Abstract

The luteal phase of the menstrual cycle is a pivotal period characterized by hormonal intricacies that lay the foundation for successful embryo implantation and early pregnancy development. Luteal phase defect (LPD), marked by abnormalities in luteal function, presents challenges that can impede reproductive outcomes. This comprehensive review article explores the role of dydrogesterone in LPD management, elucidating its mechanisms of action, evidence of efficacy, safety profile, and potential in combination therapies. Dydrogesterone, a synthetic progestogen, closely mirrors natural progesterone's actions, effectively supplementing the luteal phase and enhancing endometrial receptivity. Clinical studies demonstrate improved pregnancy rates, extended luteal phase support, and enhanced reproductive outcomes with dydrogesterone supplementation. Its favorable safety profile, minimal side effects, and reduced risk of unwanted hormonal effects contribute to its appeal. Furthermore, dydrogesterone's inclusion in international guidelines solidifies its importance in LPD management. Combination therapies, leveraging synergistic effects, offer a comprehensive approach. As gaps in knowledge persist, future research directions and personalized treatment strategies pave the way for a future where dydrogesterone stands as a beacon of hope in conquering the challenges of LPD and achieving successful reproductive outcomes.

## Introduction and background

The luteal phase, a critical component of the menstrual cycle, ensures successful reproduction by creating an optimal environment for embryo implantation and early pregnancy development. Disruptions in this phase can lead to a condition known as luteal phase defect (LPD), which has garnered significant attention within the realm of reproductive health. This review article delves into the multifaceted aspects of LPD and its management, focusing on the promising role of dydrogesterone as a therapeutic intervention [1,2].

Luteal phase defect, characterized by inadequate progesterone production and a shortened luteal phase duration, has emerged as a noteworthy concern in reproductive medicine. It is associated with compromised endometrial receptivity, hampered embryo implantation, and an increased risk of early pregnancy loss. LPD is a potential culprit behind unexplained infertility and recurrent miscarriages, prompting researchers and clinicians to investigate effective management strategies [3].

The luteal phase follows ovulation and is governed by the corpus luteum, a temporary endocrine structure formed from the remains of the ovarian follicle. Progesterone, the hormone predominantly produced during this phase, is pivotal in preparing the uterine lining for embryo attachment and nurturing the early stages of pregnancy. Imbalances or defects in this phase can result in an inadequate uterine environment, hindering the embryo's ability to implant successfully and leading to pregnancy complications [4].

Dydrogesterone, a synthetic progestogen with unique pharmacological properties, has garnered considerable attention as a potential solution for LPD management. Unlike other progestogens, dydrogesterone has structural similarities to natural progesterone, making it a more biologically compatible option for luteal phase support. Its selective progestogenic activity, combined with minimal androgenic, glucocorticoid, and mineralocorticoid effects, positions dydrogesterone as an attractive candidate for improving pregnancy outcomes in individuals with LPD [5].

This review article aims to comprehensively explore the intricate relationship between luteal phase defect and reproductive health, highlighting the crucial role of dydrogesterone as a potential therapeutic agent. By synthesizing existing literature, clinical studies, and guidelines, we seek to provide a comprehensive overview of dydrogesterone's mechanism of action, efficacy, safety profile, and potential combination therapies. Additionally, this article will emphasize the implications of dydrogesterone use in LPD management and its contribution to enhancing reproductive outcomes.

## Review

Physiology of the luteal phase

The luteal phase of the menstrual cycle plays a vital role in preparing the body for a potential pregnancy. It unfolds as a complex sequence of events orchestrated by a delicate interplay of hormones and physiological changes. This phase follows ovulation, the release of a mature egg from the ovarian follicle, and sets the stage for the critical early stages of pregnancy [6]. During the luteal phase, the once-released ovarian follicle transforms into a structure known as the corpus luteum. This transformation is accompanied by a cascade of hormonal shifts and molecular adaptations that work in concert to create an environment highly conducive to supporting a developing embryo. The corpus luteum becomes a temporary endocrine gland, producing progesterone, a hormone that holds paramount importance in establishing and maintaining the uterine lining, also known as the endometrium. This endometrial preparation is crucial for the successful implantation of a fertilized egg [7].

The hormonal shifts during this phase are orchestrated primarily by two essential hormones: progesterone and estrogen. These hormones work in tandem to stimulate the development and maintenance of the endometrial lining, making it receptive to a fertilized egg. Additionally, the increased production of progesterone acts to suppress further ovulation, preventing the release of more eggs while the body prepares for a potential pregnancy [8]. Molecular adaptations further enhance the endometrial environment for successful embryo implantation. The endometrial cells undergo changes that increase their receptivity to an embryo, facilitating attachment to the uterine wall. Blood vessels in the endometrium grow and become more permeable, nourishing the developing embryo [9].

The luteal phase represents a choreographed symphony of hormonal shifts and molecular changes. Its culmination in establishing the corpus luteum and the subsequent rise in progesterone levels transforms the uterus into an optimal environment for embryo implantation. This period of preparation and anticipation lays the foundation for early pregnancy development, making the luteal phase a critical juncture in the menstrual cycle [4].

Regular Hormonal Changes During the Luteal Phase

The luteal phase, a pivotal stage in the menstrual cycle, is distinguished by a cascade of hormonal events that orchestrate the conditions necessary for successful embryo implantation and the early stages of pregnancy. The hormone progesterone is at the heart of this phase, whose dynamic secretion is mainly orchestrated by a small but significant structure known as the corpus luteum [4]. The initiation of the luteal phase hinges on a sequence of hormonal events that begins with the release of follicle-stimulating hormone (FSH) from the anterior pituitary gland. FSH stimulates the development of a mature follicle in the ovary, eventually culminating in ovulation. This released egg is then encapsulated within the corpus luteum, a temporary endocrine gland formed from the remnants of the ruptured follicle [10]. The subsequent surge in luteinizing hormone (LH), another crucial pituitary hormone, follows the surge of FSH. LH's surge catalyzes the transformation of the follicular remnants into the corpus luteum. This event is pivotal because the corpus luteum produces progesterone, a hormone essential to sustain the uterine environment and facilitate embryo implantation.

As the corpus luteum assumes its role, it secretes progesterone in increasing amounts. This rise in progesterone levels serves a dual purpose: it prepares the uterine lining, or endometrium, for the possible arrival of an embryo, and it also feeds back to the anterior pituitary and hypothalamus to inhibit further secretion of FSH and LH. This self-regulatory mechanism is crucial in preventing the development of new ovarian follicles and maintaining the corpus luteum's integrity and functionality [11]. Suppressing FSH and LH through progesterone's feedback loop has far-reaching implications. Firstly, it prevents the ovaries from producing additional follicles, ensuring that only one dominant follicle is released during each menstrual cycle. Secondly, this suppression supports the corpus luteum's longevity, a prerequisite for producing adequate progesterone levels [12].

Establishment and Maintenance of the Corpus Luteum

After ovulation, a remarkable transformation takes place within the female reproductive system. This transformation involves the dynamic interplay between the granulosa and theca cells that were once a part of the mature follicle, creating a temporary endocrine structure known as the corpus luteum. This process, scientifically termed "luteinization," marks a crucial shift in hormonal function and lays the groundwork for potential pregnancy [13].

The granulosa and theca cells, instrumental in nurturing the maturing egg within the follicle, undergo a profound metamorphosis. Once the egg is released during ovulation, these cells change their role and structure to become luteal cells. This transformation is not merely superficial; it involves a complex biochemical cascade that fundamentally alters the cellular machinery [14].

As luteal cells, they primarily aim to secrete progesterone, a hormone of paramount importance in reproductive processes. Progesterone serves as a linchpin in orchestrating the events that lead to embryo implantation and the establishment of early pregnancy. Moreover, the corpus luteum is not a one-trick wonder; it produces progesterone and smaller quantities of estrogen, albeit in comparison to its larger counterpart, the follicle [15].

These hormonal outputs are far from trivial. Progesterone, the flagship hormone of this phase, undertakes multiple roles. It transforms the uterine lining, the endometrium, into a receptive and nurturing environment for a potential embryo. This transformation involves thickening the endometrial lining, fostering the growth of blood vessels, and promoting the secretion of nourishing substances. Essentially, the endometrium is prepared like fertile soil, ready to embrace and nourish the seed of new life [16]. Estrogen, though in smaller amounts, plays a supportive role. It helps maintain the health of the endometrial lining and ensures a harmonious environment for embryo implantation [17].

In this intricate dance of hormones, the corpus luteum holds the spotlight during the first crucial weeks of pregnancy. Its progesterone production sustains the endometrium, keeping it intact and supporting embryo attachment. However, if pregnancy does not occur, the corpus luteum's lifespan is limited. Without the signal of pregnancy, it eventually degenerates, causing progesterone levels to decline. This decline triggers the shedding of the endometrial lining, resulting in menstruation, and the cycle starts anew [3]. Transforming granulosa and theca cells into the corpus luteum showcases nature's elegant preparation for a potential pregnancy. Progesterone production and modest estrogen levels by the corpus luteum set the stage for the intricate symphony of embryo implantation and early pregnancy. It is a biological performance that underscores the remarkable precision and coordination of the female reproductive system [13].

Role of Progesterone in Endometrial Preparation and Embryo Implantation

Progesterone, an essential hormone in the menstrual cycle and early pregnancy, exerts many effects on the endometrium, orchestrating a finely tuned sequence of changes crucial for optimizing its receptivity to an embryo. Under the influence of progesterone, the endometrium undergoes a complex decidualization process. During decidualization, the endometrial stromal cells transform into specialized decidual cells. This transformation is fundamental, creating a supportive environment well-equipped to accommodate and nurture an implanting embryo [18]. This intricate process involves various structural and functional alterations within the endometrium. The decidual cells produce a range of factors, including growth factors and cytokines, which play pivotal roles in creating a welcoming milieu for embryo implantation. The heightened secretory activity of the uterine glands and the establishment of an enhanced vascular network within the endometrium are among the orchestrated changes that optimize the implantation process [19].

Progesterone's influence extends beyond structural adjustments. It acts as a natural regulator of uterine contractions, effectively suppressing them. This function is critical to prevent premature expulsion of the embryo before it has securely implanted into the endometrium. This dual role of supporting implantation and safeguarding its success by controlling uterine contractility underscores the significance of progesterone in early pregnancy [20]. Furthermore, progesterone orchestrates an intricate dance with the immune system within the uterus. It promotes a unique state of immune tolerance conducive to embryo implantation. This immunomodulatory function is pivotal, as it prevents the immune system from recognizing the embryo as a foreign entity and mounting a response that could potentially hinder implantation.

Luteal phase defect (LPD)

Luteal phase defect (LPD) represents a complex reproductive condition characterized by abnormalities in the duration and function of the luteal phase of the menstrual cycle. LPD has gained significant attention due to its potential impact on fertility outcomes and early pregnancy loss. This section delves into the nuances of LPD, including its definition, classification, underlying causes, and clinical consequences [21].

Definition and Classification of LPD

A luteal phase defect is an insufficient or inadequately maintained luteal phase, resulting in inadequate progesterone levels and impaired endometrial receptivity. This defect can manifest in different ways, such as a shortened luteal phase duration, suboptimal progesterone levels, or a combination of both. LPD can be classified into primary and secondary forms. Primary LPD occurs without any apparent underlying cause, while secondary LPD is often associated with specific conditions, such as polycystic ovary syndrome (PCOS) or thyroid disorders [22].

Causes of and Contributing Factors to LPD

The etiology of luteal phase defect (LPD) is intricate and multifaceted, with various factors contributing to its development. These factors collectively lead to disruptions in the delicate hormonal balance essential for normal luteal phase function. Common causes of LPD encompass the following: hormonal imbalances, ovarian dysfunction, stress and lifestyle factors, thyroid dysfunction, polycystic ovary syndrome (PCOS), and uterine abnormalities.

Hormonal imbalances: The orchestration of hormonal signals between the hypothalamus, pituitary gland, and ovaries is critical for successfully progressing the luteal phase. Insufficient luteinizing hormone (LH) surge, inadequate progesterone production, or impaired communication among these endocrine players can disturb the intricate hormonal interplay. Such imbalances thwart the timely formation and maintenance of the corpus luteum, a fundamental structure responsible for progesterone secretion [23].

Ovarian dysfunction: The process of follicular development and ovulation is complex, involving precise synchronization of hormonal cues. Irregularities in these processes can result in compromised corpus luteum formation, leading to reduced progesterone secretion. As progesterone is central to the endometrial preparation necessary for embryo implantation, diminished secretion can negatively impact the uterine environment's receptivity [24].

Stress and lifestyle factors: Chronic stress, excessive exercise, and maintaining a low body weight influence the hypothalamic-pituitary-ovarian axis, affecting hormonal equilibrium. These factors disrupt the finely tuned hormonal orchestration required for optimal luteal phase function. Stress-related alterations in hormone secretion, particularly cortisol, can indirectly hinder the formation and function of the corpus luteum [25].

Thyroid dysfunction: The thyroid gland plays a crucial role in hormonal regulation and metabolic processes. Abnormal thyroid function, specifically hypothyroidism, can disrupt the intricate hormonal cascade required for proper luteal phase development. Thyroid hormones influence LH and follicle-stimulating hormone (FSH) secretion, impacting ovarian function and progesterone production [26].

Polycystic ovary syndrome (PCOS): PCOS is characterized by hormonal imbalances, notably elevated androgens, and disrupted ovulation. The hormonal irregularities associated with PCOS, including elevated LH levels and reduced FSH levels, can adversely affect the establishment and maintenance of the corpus luteum, leading to insufficient progesterone secretion and compromised luteal phase function [27].

Uterine abnormalities: The uterus plays a pivotal role in successful embryo implantation. Structural abnormalities within the uterus, such as fibroids, adhesions, or congenital anomalies, can impede the establishment of a receptive endometrium. These abnormalities compromise the uterine environment's ability to support embryo implantation, regardless of the corpus luteum's function [28].

Clinical Manifestations and Impact on Fertility

Shortened menstrual cycle: A hallmark feature of LPD is a luteal phase duration that falls below the normal range, often shorter than 10 days. This abbreviated time frame poses a significant challenge to the endometrium's ability to achieve the optimal receptivity necessary for successful embryo implantation. Inadequate progesterone secretion during this limited luteal phase window impairs the transformation of the endometrium into a suitable environment for embryo attachment and growth, thus hindering the establishment of pregnancy [29].

Irregular menstrual cycles: LPD can introduce irregularities in the menstrual cycle, causing variations in the length of the cycle, the timing of ovulation, and the overall predictability of fertility windows. The inconsistency in the timing of ovulation and fertile days makes it difficult for individuals to time intercourse for conception accurately. This unpredictability further challenges family planning efforts and reduces the chances of a successful pregnancy [30].

Recurrent miscarriages: LPD is associated with an elevated risk of recurrent early pregnancy loss. The compromised endometrial support resulting from inadequate luteal phase function creates an unfavorable environment for embryo implantation and subsequent development. This deficiency in sustaining the pregnancy leads to frequent miscarriages during the early stages of gestation, causing significant emotional distress and reproductive setbacks for affected individuals [31].

Unexplained infertility: LPD can contribute to unexplained infertility, a perplexing scenario in which individuals experience difficulty achieving pregnancy despite regular ovulation and sperm parameters. The role of the luteal phase becomes apparent in such cases, as even with successful fertilization, insufficient luteal phase support can prevent the embryo from implanting and progressing [32].

Implantation failure: The ramifications of LPD extend to assisted reproductive techniques (ARTs), where repeated embryo implantation failure occurs. The inadequate endometrial receptivity stemming from LPD poses a formidable obstacle to successful procedures such as in vitro fertilization (IVF). Despite the successful creation of embryos, the compromised uterine environment diminishes the prospects of implantation, leading to repeated disappointments for individuals undergoing ART [33].

Dydrogesterone: Mechanism of action

Dydrogesterone, a synthetic progestogen, has gained significant recognition for its pivotal role in managing luteal phase defects (LPD) and addressing various reproductive conditions. Its mechanism of action is grounded in its distinctive pharmacological attributes, which distinguish it from other progestogens, rendering it an attractive choice for optimizing luteal phase function and ultimately enhancing reproductive outcomes [34].

Unique Pharmacological Characteristics

Dydrogesterone stands out due to its structural similarity to endogenous progesterone, the hormone crucial for maintaining the uterine environment during the luteal phase. Unlike some other progestogens, dydrogesterone closely mirrors the molecular structure of natural progesterone. This similarity enables dydrogesterone to interact with progesterone receptors selectively, specifically, those involved in uterine function, while minimizing interactions with other hormonal pathways [35].

Enhancing Luteal Phase Function

Dydrogesterone's primary mode of action revolves around providing exogenous progesterone-like support during the luteal phase of the menstrual cycle. By mimicking the actions of endogenous progesterone, dydrogesterone bolsters the crucial processes that occur during this phase, such as promoting decidualization and fostering a receptive endometrial lining. These actions create an environment conducive to successful embryo implantation and early pregnancy maintenance [34].

Compared with other progestogens, dydrogesterone's uniqueness stems from its selective binding and minimal interference with other hormonal receptors. In contrast, other progestogens might have nonspecific effects on androgen, glucocorticoid, or mineralocorticoid receptors, leading to potential side effects that could impact reproductive outcomes. Dydrogesterone's specificity and affinity for progesterone receptors make it an appealing option, particularly in scenarios where targeted progestogenic actions are desired without the risk of undesirable hormonal effects [36].

Improved Reproductive Outcomes

Dydrogesterone is critical in optimizing luteal phase function by offering tailored progestogenic support that closely mirrors natural progesterone's actions. This optimization, in turn, contributes to better endometrial receptivity, creating an environment conducive to embryo implantation and early pregnancy development. The enhanced reproductive outcomes observed in clinical studies underscore the significance of dydrogesterone's mechanism of action in LPD management [37].

Brief Overview of Dydrogesterone as a Progestogen

Dydrogesterone is a synthetic derivative of naturally occurring progesterone. Unlike other progestogens, dydrogesterone closely mimics endogenous progesterone's molecular structure and biological actions. This structural similarity contributes to its selectivity and efficacy in exerting progestogenic effects without significant androgenic, glucocorticoid, or mineralocorticoid activity [38].

Explanation of How Dydrogesterone Supports the Luteal Phase

Dydrogesterone's role in supporting the luteal phase is rooted in its ability to mimic the actions of progesterone, an essential hormone for sustaining early pregnancy. The luteal phase, characterized by the corpus luteum's formation after ovulation, requires optimal progesterone levels to prepare the uterine lining for embryo implantation and maintain a nurturing environment for the developing pregnancy [37]. As a synthetic progestogen, dydrogesterone is an exogenous source of progesterone-like support during this critical phase. By providing additional progesterone-like activity, dydrogesterone ensures that the endometrium becomes adequately receptive for successful embryo attachment. This is achieved through multiple mechanisms [39].

Firstly, dydrogesterone supports the process of decidualization, which involves the transformation of the uterine lining into a specialized tissue that is well-suited to support embryo implantation. This process requires an orchestrated interplay of hormonal signals, with progesterone playing a central role. Dydrogesterone's ability to replicate this progesterone-driven signal ensures that the endometrium is adequately prepared to receive the embryo [40]. Secondly, dydrogesterone enhances endometrial receptivity by promoting the growth and development of uterine glands and blood vessels. This fosters an environment conducive to embryo implantation, allowing for successful interaction between the embryo and the uterine lining [8]. Furthermore, dydrogesterone addresses potential luteal phase insufficiencies that may arise due to various underlying factors. It ensures that the progesterone levels necessary for a supportive luteal phase are maintained, preventing premature shedding of the uterine lining and increasing the chances of embryo implantation.

A notable advantage of dydrogesterone is its selectivity for progesterone receptors, which minimizes interference with other hormonal pathways. Unlike some progestogens, dydrogesterone's actions target progesterone-related processes without significantly activating androgenic, glucocorticoid, or mineralocorticoid receptors. This selectivity preserves the hormone's desired effects on the endometrium and reduces the risk of unwanted side effects [36].

Comparison of Dydrogesterone With Other Progestogens

Dydrogesterone distinguishes itself from other progestogens through its remarkable structural similarity to natural progesterone. This unique feature sets the stage for a profound impact on its mode of action and clinical implications. By closely emulating the biological actions of endogenous progesterone, dydrogesterone becomes a standout option for luteal phase support, addressing the intricacies of reproductive health with precision [41].

The structural resemblance between dydrogesterone and natural progesterone endows it with a heightened ability to interact with progesterone receptors in a manner akin to the body's hormonal processes. This enables dydrogesterone to exert targeted progestogenic effects, fostering endometrial receptivity and facilitating a suitable environment for embryo implantation and early pregnancy development. Its compatibility with the body's natural hormonal pathways ensures seamless integration of its actions, enhancing its efficacy in managing luteal phase defects [42].

In contrast, certain other progestogens may carry the risk of nonspecific effects on various hormonal receptors, such as androgen, glucocorticoid, or mineralocorticoid receptors. This nonspecificity can give rise to potential side effects that affect reproductive outcomes. For instance, androgenic side effects could disrupt hormonal balance and hinder the delicate processes for successful conception and pregnancy [42].

This comparison underscores dydrogesterone's pharmacological superiority, especially when precise, targeted progestogenic effects are desired without interfering with other hormonal pathways. Dydrogesterone's potential to offer fewer side effects and a more favorable tolerability profile makes it an appealing and prudent choice for managing luteal phase defects. This advantage can have far-reaching implications, optimizing reproductive success and enriching the quality of care for individuals navigating the complexities of fertility and pregnancy [43].

Evidence of Dydrogesterone Efficacy

The efficacy of dydrogesterone in managing luteal phase defect (LPD) has been extensively investigated through clinical studies and trials. This section comprehensively reviews the evidence, highlighting critical findings on pregnancy rates, luteal phase support, and overall reproductive outcomes [39].

Numerous clinical studies and randomized controlled trials (RCTs) have explored dydrogesterone's role in LPD management. These trials have evaluated its effectiveness in diverse patient populations, including those undergoing assisted reproductive techniques (ART) and those with idiopathic infertility. Studies have employed various routes of administration, such as oral and vaginal, to investigate the optimal way to deliver dydrogesterone for luteal phase support [44].

Pregnancy rates: Clinical evidence suggests that dydrogesterone supplementation positively impacts pregnancy rates in individuals with LPD. Studies have reported improved implantation rates and higher clinical pregnancy rates among patients receiving dydrogesterone compared to placebo or other progestogens. This indicates that dydrogesterone's support of the luteal phase contributes to a more conducive environment for embryo implantation and early pregnancy development [45].

Luteal phase support: Dydrogesterone effectively extends the luteal phase, thereby addressing one of the critical abnormalities in LPD. The hormone's progestogenic actions support the endometrium during this critical period, enhancing endometrial receptivity and preventing premature shedding. This is particularly important for individuals with LPD, as an adequately maintained luteal phase is essential for sustaining pregnancy [37].

Reproductive outcomes: Studies have shown that dydrogesterone supplementation improves reproductive outcomes, especially in patients with LPD. These outcomes include increased live birth rates and decreased miscarriage rates. Dydrogesterone enhances overall reproductive success by optimizing the uterine environment and fostering embryo implantation [45].

Administration and dosage

Dydrogesterone's efficacy in managing luteal phase defect (LPD) extends to its various forms of administration and dosage regimens. This section explores the different available forms of dydrogesterone, outlines recommended dosage regimens for LPD treatment, and emphasizes the importance of considering patient preferences and clinical scenarios when choosing the administration route [46].

Different Forms of Dydrogesterone Available

Oral tablets: The availability of oral dydrogesterone tablets offers a convenient and widely adopted option for LPD management. These tablets can be easily administered and absorbed through the digestive system, enabling efficient hormone delivery. This systemic approach ensures that the necessary levels of dydrogesterone are reached in the bloodstream, facilitating its actions in supporting the luteal phase and enhancing endometrial receptivity. The ease of use and familiarity associated with oral administration contribute to the popularity of this form of dydrogesterone, making it a practical choice for patients and healthcare providers alike [40].

Vaginal suppositories or tablets: Vaginal administration of dydrogesterone introduces a distinct approach that capitalizes on the local route for hormone delivery. Using suppositories or tablets placed directly into the vagina, dydrogesterone can exert its effects locally within the uterine environment. This method allows for direct endometrial exposure, which could enhance the hormone's impact on endometrial receptivity. Furthermore, the local delivery might lead to decreased systemic side effects, as the hormone's systemic circulation might be limited. This approach is particularly relevant for individuals seeking to minimize potential systemic effects or with specific medical conditions that warrant a more localized intervention [5].

Sublingual tablets: Sublingual administration introduces another avenue for dydrogesterone delivery. Sublingual tablets are placed under the tongue, where they rapidly dissolve and are absorbed directly into the bloodstream through the rich network of blood vessels in the area. This approach offers a potential balance between systemic and local effects. The quick absorption into the bloodstream allows for efficient systemic distribution of dydrogesterone while avoiding the digestive system, which could reduce the risk of metabolism or degradation. Sublingual administration could be especially beneficial for individuals who require precise and rapid control over their hormone levels during the luteal phase [47].

Recommended Dosage Regimens for LPD Treatment

Oral tablets: In the context of oral administration, standard dosage ranges typically span from 10 to 40 mg of dydrogesterone daily. This dosage is often divided into two or more doses to maintain stable progesterone levels throughout the luteal phase. The treatment regimen generally encompasses the second half of the menstrual cycle, corresponding to the luteal phase. This approach ensures continuous progestogenic support during the critical period for embryo implantation and early pregnancy establishment [5].

Vaginal suppositories or tablets: Vaginal administration offers a targeted route for delivering dydrogesterone directly to the uterine environment. Dosage recommendations for vaginal suppositories or tablets typically range from 200 to 400 mg per day. This dosage is distributed into two separate doses to ensure sustained progesterone exposure to the endometrium. By delivering dydrogesterone locally, this approach optimizes endometrial receptivity and enhances support for embryo implantation and pregnancy maintenance [5].

Sublingual tablets: Sublingual administration involves placing dydrogesterone tablets under the tongue for rapid absorption into the bloodstream. Sublingual dosage regimens often mirror oral administration, ranging from 10 to 40 mg per day. Like oral dosing, sublingual doses can be divided to maintain consistent progesterone levels during the luteal phase. This approach offers an alternative systemic delivery method while minimizing potential gastrointestinal effects [39].

Consideration of Patient Preferences and Clinical Scenarios

Choosing the optimal administration route of dydrogesterone involves carefully evaluating various factors, including patient preferences, clinical situations, and potential side effects. The individual's comfort and treatment adherence are pivotal in this decision-making process. Patients often have unique preferences regarding the method of administration, and taking these preferences into account can significantly enhance their commitment to the treatment plan. Moreover, the presence of specific clinical conditions further shapes the choice of administration route [48].

In assisted reproductive techniques (ARTs), the administration route can be tailored to the specific treatment plan and the clinician's expertise. In scenarios where optimizing endometrial receptivity is paramount, such as in vitro fertilization (IVF) cycles, the choice of administration route gains even more significance. The vaginal route, for instance, can be favored in these cases due to its direct access to the uterine environment. This approach aims to enhance the interaction between dydrogesterone and the endometrium, promoting optimal endometrial conditions for successful embryo implantation [49].

Ultimately, the decision regarding the administration route of dydrogesterone is nuanced, shaped by a delicate balance of patient preferences, clinical considerations, and the overarching treatment strategy. Tailoring the route to suit individual needs can enhance treatment adherence and contribute to the overall success of LPD management and reproductive outcomes [50].

Safety and tolerability

In reproductive medicine, the careful balance between therapeutic effectiveness and patient well-being takes center stage. The paramount significance of safety and tolerability cannot be understated, particularly when addressing conditions such as luteal phase defect (LPD). As a potential solution in LPD management, dydrogesterone undergoes a rigorous evaluation of its safety profile and side effects, serving as a linchpin in its utilization [51].

Understanding Safety and Side Effects

The safety profile of dydrogesterone is a cornerstone of its acceptance in clinical practice. Identifying and quantifying potential side effects are essential in making informed treatment decisions. These side effects, often transient and mild, can include gastrointestinal discomfort, breast tenderness, and occasional headaches. Dydrogesterone's profile sets it apart from other progestogens, demonstrating a minimal risk of unwanted hormonal effects, including androgenic or glucocorticoid-related complications [35].

Comparative Evaluation

The comparative analysis of dydrogesterone with other progestogens brings forth its unique attributes. Unlike certain progestogens associated with undesirable side effects, dydrogesterone's structural resemblance to natural progesterone engenders a more favorable tolerability profile. The selective targeting of progestogenic actions minimizes potential disruptions in other hormonal pathways. This comparative advantage positions dydrogesterone as an appealing option, particularly when considering patient comfort and treatment adherence [52].

Tailoring to Special Patient Populations

The essence of personalized medicine resonates in the tailored attention required for special patient populations. Individuals who are pregnant or breastfeeding or possess specific medical conditions necessitate a nuanced approach to dydrogesterone treatment. While its safety remains, special care ensures that treatment aligns with individual circumstances. Addressing the needs of these specific groups enhances the overall therapeutic experience and optimizes treatment outcomes [53].

Overview of Safety Profile and Side Effects

As a therapeutic option for luteal phase defect (LPD) management, dydrogesterone boasts a reassuring safety profile that is generally well-tolerated by patients. Individuals undergoing dydrogesterone treatment commonly experience mild and transient side effects, which, if present, tend to be manageable [43].

One of the potential side effects associated with dydrogesterone treatment is gastrointestinal upset. Some individuals might encounter minor disturbances in the gastrointestinal tract, such as bouts of nausea or bloating. These effects, while noticeable, are often temporary and generally do not escalate into severe discomfort [35].

Breast tenderness is another noteworthy side effect that could arise during dydrogesterone treatment. This occurrence is attributed to the hormone's progestogenic activity. Dydrogesterone's action on progesterone receptors in breast tissue can increase sensitivity and mild breast discomfort. It is important to note that this symptom is typically manageable and tends to diminish over time as the body adapts to the hormonal changes [54].

Reports of headaches have been sporadically associated with dydrogesterone use, although their frequency is generally low. These headaches are usually mild and infrequent and tend to abate as treatment progresses. It is important to differentiate between incidental headaches and those directly linked to dydrogesterone use, as headaches can stem from various factors [55].

Its favorable progestogenic selectivity is an important differentiating factor that sets dydrogesterone apart from certain other progestogens. This means that dydrogesterone's mechanism of action is primarily targeted at progesterone receptors, minimizing the risk of triggering androgenic, glucocorticoid, or mineralocorticoid side effects commonly observed with other progestogens. This selectivity contributes to a more comfortable experience for patients undergoing dydrogesterone treatment [36].

Comparison of Dydrogesterone's Safety With Other Progestogens

Dydrogesterone's safety profile stands out due to its structural similarity to natural progesterone. Unlike other progestogens, dydrogesterone's unique chemical structure minimizes the risk of androgenic or other unwanted hormonal effects. This reduced risk of side effects positions dydrogesterone as an appealing option for individuals seeking luteal phase support with minimal disruptions to their overall well-being [56].

Special Considerations for Specific Patient Populations

Pregnancy: Dydrogesterone plays a significant role in supporting the luteal phase during the early stages of pregnancy, particularly in cases where a history of recurrent miscarriages or threatened abortion exists. These situations are often associated with inadequate progesterone levels, essential for maintaining the uterine environment conducive to embryo implantation and sustaining pregnancy. Dydrogesterone's safety profile makes it a suitable choice for this purpose. Its structural resemblance to natural progesterone allows it to provide supplemental support to the corpus luteum, ensuring sustained progesterone levels critical for preserving endometrial receptivity and early pregnancy support. By maintaining a nurturing uterine environment, dydrogesterone offers a potential solution to the challenges faced by individuals with a history of pregnancy loss or threatened abortion [57].

Breastfeeding: Dydrogesterone's compatibility with breastfeeding is essential for individuals seeking LPD management postpartum. The minimal transfer of dydrogesterone into breast milk makes it generally safe for use during lactation. This characteristic suggests that the potential impact on breastfeeding infants is limited, minimizing any concerns about exposure to the medication. However, it is important to note that individual circumstances can vary, and medical advice should guide treatment decisions. Healthcare professionals should evaluate the overall health of both the mother and the breastfeeding infant before recommending dydrogesterone. This cautious approach ensures that treatment choices prioritize the well-being of both mother and child [58].

Medical conditions: Patients with specific medical conditions require careful consideration when using dydrogesterone for LPD management. Conditions such as liver dysfunction or a history of thromboembolic disorders can influence the body's metabolism and medication response. In these cases, physicians should exercise vigilance and thorough evaluation before prescribing dydrogesterone. Liver function tests may be recommended to assess the drug's potential impact on hepatic health. Additionally, individuals with a history of thromboembolic disorders, which involve the formation of blood clots, need close monitoring due to the potential interaction of dydrogesterone with the body's coagulation system. These precautions underscore the importance of tailoring treatment to each patient's unique medical history, ensuring that the benefits of dydrogesterone outweigh any potential risks associated with underlying medical conditions [59].

Combination therapies

The intricacies of managing luteal phase defects (LPD) call for a comprehensive strategy that addresses the multifaceted challenges posed by this condition. Recognizing that LPD arises from a complex interplay of hormonal dysregulation, endometrial abnormalities, and other contributing factors, the approach to treatment goes beyond a single intervention. Combination therapies, which involve the simultaneous use of different medications, emerge as a strategic avenue to tackle LPD's complexities and optimize reproductive outcomes [60].

Dydrogesterone's Role in Combination Therapies

Dydrogesterone, as a powerful player in LPD management, can synergize with other medications to create a more effective and holistic treatment approach. This might involve the integration of dydrogesterone with other interventions commonly employed in fertility treatments, such as gonadotropins and gonadotropin-releasing hormone (GnRH) agonists [61].

Rationale Behind Combination Therapies

The rationale for combination therapies stems from the need to target LPD from various angles, each aligned with its underlying mechanisms. While dydrogesterone provides luteal support and enhances endometrial receptivity, medications such as gonadotropins stimulate follicular development, and GnRH agonists control hormonal surges. By combining these interventions, clinicians aim to create a harmonious hormonal environment that optimizes the chances of successful embryo implantation and early pregnancy maintenance [62].

Synergistic Effects

The real power of combination therapies lies in the potential for synergistic effects. Dydrogesterone's ability to enhance endometrial receptivity can work with gonadotropin-induced follicular growth, ensuring that a well-prepared endometrium welcomes a healthy embryo. Similarly, GnRH agonists can prevent premature luteolysis, extending the supportive environment for the developing pregnancy. When these interventions are strategically combined, the outcomes can be greater than the sum of their individual effects [63].

Clinical Customization

Combination therapies are not one-size-fits-all but tailored to each patient's unique circumstances. The choice of medications, dosages, and timing is guided by factors such as the severity of LPD, the patient's medical history, and the specifics of the treatment plan. This customization ensures that the therapy aligns perfectly with the patient's needs, optimizing the chances of achieving a successful pregnancy [64].

Exploration of Combination Therapies

Combination therapies involving dydrogesterone are strategic approaches that harness the distinct characteristics of different interventions to enhance luteal phase support and optimize reproductive success synergistically. In reproductive medicine, the luteal phase is pivotal in creating a receptive uterine environment for embryo implantation and early pregnancy establishment. Luteal phase defect (LPD), marked by inadequate progesterone levels and compromised endometrial receptivity, can impede these crucial processes [40].

To address the multifaceted challenges of LPD, researchers and clinicians have explored the potential benefits of combining dydrogesterone with other treatment modalities. These combination therapies capitalize on the strengths of each intervention to create a harmonious and supportive reproductive environment [65].

One such combination involves integrating dydrogesterone with gonadotropins within the context of assisted reproductive techniques (ARTs). Gonadotropin stimulation is routinely employed to induce follicular development, ensuring the availability of mature eggs for fertilization. However, this process could disrupt the natural hormonal balance of the luteal phase, jeopardizing the endometrial environment required for successful implantation. By supplementing dydrogesterone during the luteal phase, practitioners aim to provide essential support that sustains the endometrium and maintains the hormonal milieu conducive to embryo attachment and development [66].

Another promising combination involves the integration of dydrogesterone with gonadotropin-releasing hormone (GnRH) agonists. GnRH agonists are utilized in controlled ovarian stimulation to prevent premature luteinizing hormone (LH) surges that could result in the premature release of eggs. While this strategy optimizes follicular development, it may inadvertently compromise the luteal phase by suppressing endogenous LH surges required for corpus luteum function. By supplementing with dydrogesterone, which exhibits progestogenic support, the goal is to ensure a sustained and adequate luteal phase that maintains endometrial receptivity and prevents early luteolysis [67].

In both scenarios, the synergy of dydrogesterone with other interventions aims to address the unique challenges posed by LPD and assisted reproductive techniques. By strategically combining treatments, clinicians seek to create a holistic approach that encompasses both follicular and luteal phase support, ultimately enhancing the chances of successful embryo implantation, pregnancy establishment, and overall reproductive success. As research continues to illuminate the intricacies of combination therapies, the potential for further refining LPD management grows, offering renewed hope to individuals navigating the complexities of fertility challenges.

Rationale for combined treatments and potential synergistic effects

Elaboration on Combination Therapies and Rationale

The rationale behind combining therapies in managing luteal phase defect (LPD) is deeply rooted in this complex and multifaceted condition. LPD can arise from a convergence of various factors, ranging from hormonal imbalances to disruptions in endometrial receptivity. Recognizing this intricate interplay, combination therapies harness different interventions' strengths to create a synergistic effect that yields improved treatment outcomes [62].

Comprehensive luteal phase support: Combination therapies aim to provide comprehensive support to the luteal phase of the menstrual cycle, a critical period for successful embryo implantation and early pregnancy development. By addressing multiple aspects of this phase, such as progesterone levels and endometrial receptivity, these therapies create an environment conducive to optimal reproductive outcomes. This multifaceted approach ensures that all elements necessary for successful embryo implantation are nurtured, enhancing the chances of a viable pregnancy [68].

Minimized risks of luteal phase insufficiency: The multifactorial nature of LPD means that various mechanisms can be at play leading to inadequate luteal phase function. Combination therapies take a holistic stance by targeting these diverse mechanisms simultaneously. These therapies reduce the risk of luteal phase insufficiency by addressing the contributing factors comprehensively. This, in turn, minimizes the chances of implantation failure and early pregnancy loss, allowing for more successful reproductive outcomes [62].

Clinical guidelines and recommendations

Including dydrogesterone in international fertility and gynecology guidelines represents a significant milestone in recognizing its role as an effective intervention for managing luteal phase defects (LPD). These guidelines serve as authoritative references that offer clinicians evidence-based recommendations, providing valuable insights and strategies to enhance LPD management [49].

Dydrogesterone, a synthetic progestogen with structural similarities to natural progesterone, has garnered the attention of leading organizations and societies in fertility and gynecology. These respected bodies have meticulously evaluated the available scientific evidence and clinical data surrounding dydrogesterone's efficacy in addressing LPD. As a result, they have acknowledged its utility and explicitly endorsed its use in managing this reproductive condition [5].

These guidelines are developed collaboratively by experts in the field, often involving rigorous reviews of relevant research, clinical trials, and expert consensus. The endorsement of dydrogesterone within these guidelines signifies a consensus within the medical community regarding its value as a treatment option for LPD. Such recognition lends credibility to dydrogesterone's effectiveness and encourages its integration into clinical practice [69].

For healthcare practitioners, these guidelines offer a structured framework for LPD management, with clear recommendations on when and how to consider dydrogesterone as part of the treatment plan. They help guide decisions about administration routes, dosage regimens, and patient selection. Clinicians can feel confident choosing to include dydrogesterone in their therapeutic approach, knowing their decision aligns with the latest evidence-based guidelines established by authoritative organizations.

Furthermore, these guidelines serve as a valuable educational resource for medical professionals, providing a consolidated and up-to-date summary of the current understanding of LPD and its management. They empower clinicians to make informed decisions, tailor treatment plans to individual patient needs, and optimize outcomes for those seeking to overcome the challenges posed by luteal phase defects [70]. In essence, including dydrogesterone in international fertility and gynecology guidelines signifies the culmination of rigorous scientific evaluation, consensus-building, and expert endorsement. This recognition underscores dydrogesterone's significance as a valuable tool in the arsenal of treatments for LPD, reassuring clinicians and patients that the highest standards of medical practice support its use.

Recommended Approaches to LPD Management With Dydrogesterone

Diagnosis: Accurate diagnosis of luteal phase defect (LPD) forms the foundation of effective management. This involves a comprehensive clinical assessment, including evaluating menstrual cycle characteristics, hormonal levels (particularly progesterone), and potential contributing factors. In cases where clarity is required, an endometrial biopsy might be performed to assess endometrial receptivity and luteal phase adequacy. This thorough diagnosis enables tailored interventions to address the specific challenges of LPD [71].

Administration route and dosage: Once diagnosed, the choice of administration route and dosage regimen of dydrogesterone is crucial. Patient preferences, clinical considerations, and the severity of LPD influence this decision. The administration routes, such as oral, vaginal, or sublingual, offer different benefits and considerations. Similarly, the appropriate dosage depends on factors such as the individual's hormonal profile, the desired luteal phase length, and the treatment context [72].

Monotherapy or combination: The decision to use dydrogesterone as monotherapy or in combination with other interventions depends on individual patient needs and treatment plans. Dydrogesterone's versatile role as a progestogen allows it to be integrated into various therapeutic strategies. For instance, assisted reproductive techniques (ARTs) might be combined with gonadotropins or GnRH agonists to provide comprehensive luteal phase support and enhance the success of embryo implantation [73].

Assisted reproductive techniques (ARTs): Dydrogesterone's significance in ART protocols is particularly notable. Integrating dydrogesterone into these protocols helps create an optimal environment for embryo implantation and early pregnancy development. By extending and supporting the luteal phase, dydrogesterone is critical in maintaining endometrial receptivity, a pivotal factor in ART success [74].

Monitoring and adjustments: The response to dydrogesterone treatment should be closely monitored through regular hormonal levels and clinical outcomes assessments. Adjustments to the treatment plan might be necessary based on individual variations in response, ensuring that the luteal phase remains adequately supported throughout the treatment cycle [75].

Patient education: Educating patients about the rationale behind dydrogesterone treatment is essential for fostering understanding and adherence. Patients should be informed about the potential benefits, possible side effects (generally mild and transient), and the importance of adhering to the prescribed treatment regimen. Patient education empowers individuals to participate in their care actively and enhances treatment outcomes [76].

Future directions and research needs

Long-Term Outcomes

The need for further investigation into the long-term effects of dydrogesterone on reproductive outcomes, encompassing parameters such as live birth rates and pregnancy complications, arises as a crucial gap in current knowledge. Understanding the sustained impact of dydrogesterone treatment beyond the immediate luteal phase could provide valuable insights into its role in ensuring successful pregnancies [38].

Optimal Administration Route and Dosage

Determining the most effective administration route and dosage regimen for different patient populations and varying clinical scenarios is another gap. Tailoring treatment approaches based on individual characteristics and LPD severity is imperative for optimizing outcomes, and research in this area could yield personalized and precise interventions [77].

Combination Therapies

While dydrogesterone has been established as effective, more research is warranted to explore its potential synergistic effects when combined with other interventions. Investigating the ideal combinations of dydrogesterone with treatments such as gonadotropins or GnRH agonists could offer a comprehensive approach to LPD management, enhancing treatment outcomes [35].

Underlying Mechanisms

The mechanisms through which dydrogesterone exerts its beneficial effects on luteal phase function and endometrial receptivity remain partially understood. In-depth research into the underlying mechanisms could unveil the pathways through which dydrogesterone contributes to successful embryo implantation and early pregnancy support [37].

Suggestions for Future Research Directions

Randomized controlled trials (RCTs): To strengthen the evidence base, conducting large-scale RCTs with robust methodologies is recommended. These trials could provide a more definitive understanding of dydrogesterone's efficacy, particularly in comparison to other progestogens or alternative interventions.

Comparative studies: Comparative studies that evaluate different administration routes of dydrogesterone and compare them with other progestogens could help delineate the optimal treatment approach. Such studies would guide clinicians in selecting the most effective and patient-friendly options.

Impact on IVF outcomes: Given the prevalence of assisted reproductive techniques, focusing research on dydrogesterone's impact on outcomes in vitro fertilization (IVF) cycles is warranted. Investigating parameters such as implantation, pregnancy, and live birth rates could shed light on its role in optimizing IVF success.

Potential Areas of Improvement

Patient education: Enhancing patient education regarding LPD and the benefits of dydrogesterone could substantially improve treatment adherence and outcomes. Educated patients are more likely to actively participate in their treatment plans, leading to more successful management.

Personalized treatment: The development of predictive models that consider patient characteristics and relevant biomarkers holds the potential to guide personalized dydrogesterone treatment approaches. Tailoring treatments to individual needs could maximize the chances of success.

Long-term safety: Investigating the long-term safety of dydrogesterone is crucial, particularly its potential effects on maternal and fetal health. A comprehensive assessment of its risk-benefit profile over extended periods would provide valuable insights into its role in LPD management.

## Conclusions

In the intricate landscape of reproductive health, the luteal phase is a critical period that orchestrates the delicate interplay between hormonal dynamics and endometrial receptivity. Luteal phase defect (LPD) casts a shadow on this harmonious process, introducing complexities that can compromise embryo implantation, early pregnancy development, and overall reproductive success. This comprehensive review article has delved into the multifaceted world of LPD, exploring its definition, pathophysiology, clinical manifestations, and impact on fertility. Within this context, dydrogesterone emerges as a beacon of hope, offering a solution that addresses the underlying deficiencies of the luteal phase. Its structural similarity to natural progesterone, selectivity, and favorable pharmacological profile have positioned it as a frontline contender in LPD management. We have traversed the landscape of dydrogesterone's mechanism of action, understanding how its progestogenic support enhances endometrial receptivity and sustains early pregnancy. The evidence from clinical studies underscores its efficacy, with improved pregnancy rates, extended luteal phase support, and enhanced reproductive outcomes. Safety and tolerability are paramount in any therapeutic journey, and dydrogesterone also shines here, with minimal side effects and a reduced risk of unwanted hormonal effects. Patient preferences and special considerations for unique populations provide a personalized touch to its administration, enhancing patient experience and outcomes. As dydrogesterone finds its place within international guidelines, its role in LPD management is validated. Combination therapies, guided by the wisdom of synergistic effects, hold the potential to usher in a new era of comprehensive care, addressing the multifaceted challenges of LPD with precision.
